# The BMP2 nuclear variant, nBMP2, is expressed in mouse hippocampus and impacts memory

**DOI:** 10.1038/srep46464

**Published:** 2017-04-18

**Authors:** Ryan D. Cordner, Lindsey N. Friend, Jaime L. Mayo, Corinne Badgley, Andrew Wallmann, Conrad N. Stallings, Peter L. Young, Darla R. Miles, Jeffrey G. Edwards, Laura C. Bridgewater

**Affiliations:** 1 Department of Microbiology and Molecular Biology, Brigham Young University, Provo, Utah, USA; 2 Department of Physiology and Developmental Biology, Brigham Young University, Provo, Utah, USA

## Abstract

The novel nuclear protein nBMP2 is synthesized from the BMP2 gene by translational initiation at an alternative start codon. We generated a targeted mutant mouse, nBmp2NLS^tm^, in which the nuclear localization signal (NLS) was inactivated to prevent nuclear translocation of nBMP2 while still allowing the normal synthesis and secretion of the BMP2 growth factor. These mice exhibit abnormal muscle function due to defective Ca^2+^ transport in skeletal muscle. We hypothesized that neurological function, which also depends on intracellular Ca^2+^ transport, could be affected by the loss of nBMP2. Age-matched nBmp2NLS^tm^ and wild type mice were analyzed by immunohistochemistry, behavioral tests, and electrophysiology to assess nBMP2 expression and neurological function. Immunohistochemical staining of the hippocampus detected nBMP2 in the nuclei of CA1 neurons in wild type but not mutant mice, consistent with nBMP2 playing a role in the hippocampus. Mutant mice showed deficits in the novel object recognition task, suggesting hippocampal dysfunction. Electrophysiology experiments showed that long-term potentiation (LTP) in the hippocampus, which is dependent on intracellular Ca^2+^ transport and is thought to be the cellular equivalent of learning and memory, was impaired. Together, these results suggest that nBMP2 in the hippocampus impacts memory formation.

Our group reported the existence of a novel alternative nuclear form of bone morphogenetic protein 2, called nBMP2^1^. The conventional form of the protein, BMP2, is a secreted growth factor that affects a wide variety of developmental processes including axis formation, central nervous system development, neural crest formation, limb patterning, heart and vascular development, skeletal development, and muscle development^2^,^3^,^4^,^5^,^6^,^7^,^8^. The variant form, nBMP2, is synthesized from an alternative downstream start codon, which eliminates the N-terminal endoplasmic reticulum signal peptide and thus prevents the protein from entering the secretory pathway. Instead, nBMP2 is translated in the cytoplasm and then translocated to the nucleus by means of an embedded bipartite nuclear localization signal (NLS) (Fig. 1)^1^.

To investigate the function of nBMP2 in the nucleus without disrupting secreted BMP2, we knocked in a three-amino acid substitution mutation in the NLS to generate the nBmp2NLS^tm^ mouse^9^. The targeted mutation disrupts nuclear localization of nBMP2 while retaining normal secreted BMP2 growth factor synthesis and function^9^,^10^. Homozygous nBmp2NLS^tm^ mice are normal in size and morphology. But whereas wild type mice show expression of nBMP2 in the nuclei of skeletal muscle myotubes, mutant mice do not. The absence of nBMP2 in skeletal muscle nuclei results in slowed relaxation of skeletal muscle after stimulated contractions, consistent with impaired re-uptake of Ca^2+^ from the cytoplasm into the sarcoplasmic reticulum at the end of each contraction cycle^9^. Homozygous nBmp2NLS^tm^ mice also show an impaired secondary immune response to systemic bacterial infection, which might similarly arise from defective intracellular Ca^2+^ signaling during the process of immune cell activation or differentiation^11^.

Hippocampal synaptic plasticity, the neurochemical foundation of learning and memory, is also mediated by intracellular Ca^2+^ transport^12^,^13^,^14^,^15^. We hypothesized, therefore, that if nBMP2 was found to be expressed in hippocampus of wild type mice, learning and memory could be impaired in nBmp2NLS^tm^ mutant mice. Immunohistochemistry demonstrated that nBMP2 is indeed expressed in the hippocampus of wild type mice. The novel object recognition task suggested memory impairment in the mutant mice, and electrophysiology experiments demonstrated decreased long-term potentiation (LTP). Collectively, these experiments demonstrated that exclusion of nBMP2 from the nucleus leads to impaired memory in nBmp2NLS^tm^ mice.

## Results

### nBMP2 is expressed in the CA1 region of the hippocampus

**Figure 2:**
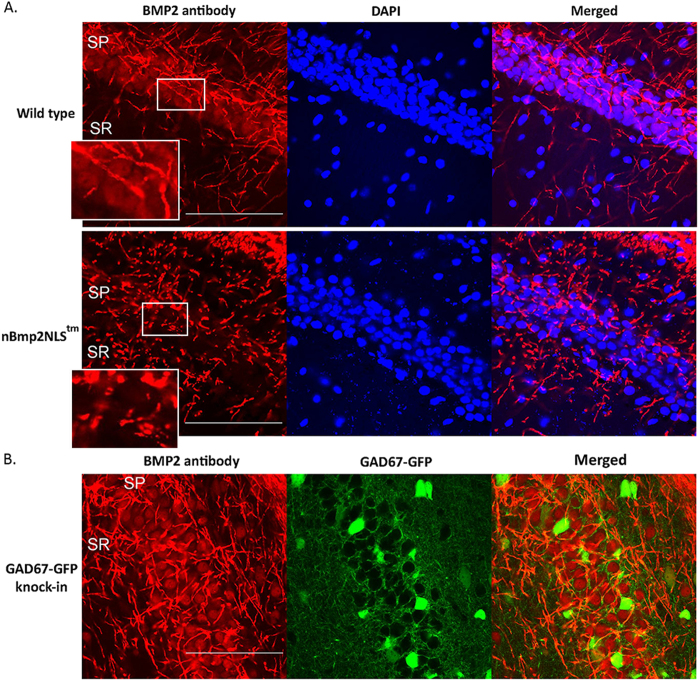
nBMP2 is expressed in the nuclei of wild type but not mutant CA1 region hippocampal neurons. (**A**) Brain sections from wild type and nBmp2NLS^tm^ mice were stained with fluorescently tagged anti-BMP2 antibody (red) and nuclei were stained with DAPI (blue). The CA1 region of the hippocampus is shown. High magnification insets of the highlighted region illustrate red BMP2 labeling in the nuclei of pyramidal cell soma of the stratum pyramidale in wild type mice, which is absent in the nBmp2NLS^tm^ mice. BMP2 labeling of neuronal projections is present in both wild type and mutant mice, confirming that the targeted mutation selectively eliminated only the nuclear variant of the protein. (**B**) Brain sections from GAD67-GFP fusion knock-in mice were stained with fluorescently tagged anti-BMP2 antibody (red). All sections were imaged using laser confocal microscopy. Scale bars indicate 100 μm. SP: stratum pyramidale; SR: stratum radiatum.

Because intracellular Ca^2+^ signaling in the hippocampus is central to object recognition memory, short- and long-term memory, spatial memory, and navigation^16^,^17^,^18^,^19^, we performed immunofluorescence staining on 2-month-old mice to determine whether nBMP2 is expressed in the hippocampus. We observed anti-BMP2 antibody staining of nuclei in the hippocampal CA1 pyramidal cell layer in wild type mice (Fig. 2A, top panel) but not mutant mice (Fig. 2A, bottom panel). Immunohistochemical staining was quantified by analysis of mean grayscale intensity of the nuclei in each image. CA1 cell nuclei from wild type mice showed significantly (p < 0.05) greater labeling intensity than nBmp2NLS^tm^ nuclei (1,814 ± 131.9 versus 294.9 ± 75 mean grayscale intensity; n = 5 and 4 mice, respectively), with nBmp2NLS^tm^ nuclei labeling only slightly more intensely than background. In addition, using a cut-off of three times the average background pixel intensity to denote positive labeling, we determined that 92.2% of pyramidal cell nuclei in wild type mice were positive for nBMP2 compared to 1.5% in nBmp2NLS^tm^ mice.

Neuronal projections showed BMP2 staining throughout the CA1 in both wild type and mutant mice, providing additional evidence that the nBmp2NLS^tm^ mutation selectively eliminates only the nuclear variant of BMP2, nBMP2.

As a second control, we also performed immunofluorescence staining on GAD67-GFP knock-in mice, which express GFP in a large percentage of inhibitory GABAergic hippocampal interneurons^20^. These results confirmed nBMP2 expression in the CA1 pyramidal cell nuclei from a different mouse strain and at a younger age (19 days) (Fig. 2B). In addition, these results revealed that 87.2% of GAD67-containing GABA neurons express nBMP2 in the nucleus, although their staining intensity was only two-thirds that of pyramidal cell nuclei (1,204 ± 68.8 mean grayscale intensity; n = 4 mice). Preabsorption of the antibody with BMP2 peptide eliminated staining of CA1 nuclei and neuronal projections in wild type, nBmp2NLS^tm^, and GAD67-GFP mice (not shown).

### Novel object recognition is reduced in nBmp2NLS^tm^ mice

**Figure 3:**
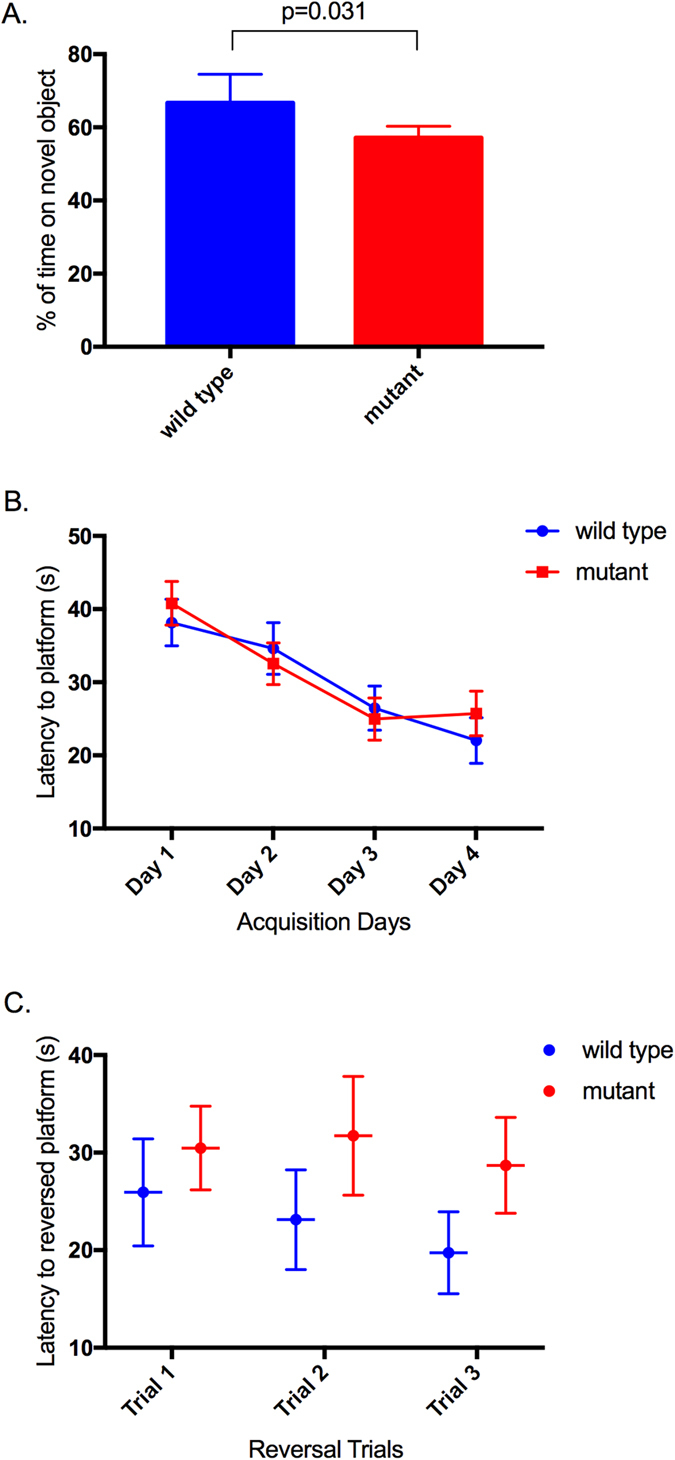
nBmp2NLS^tm^ mice exhibit object memory deficits in the novel object recognition task. (**A**) Six-month-old wild type and mutant mice were tested in the novel object recognition task. Each mouse was allowed to explore the open-field arena and its objects for 10 minutes. 90 minutes later, one of the objects was replaced with a novel object and each mouse was placed in the arena for 10 more minutes. Results are expressed as (time spent interacting with the novel object/total time spent interacting with the novel and familiar objects) × 100 ± SEM and include the results of 7 different trials. (**B**,**C**) Six-month-old wild type and mutant mice were tested in the Morris water maze. (**B**) Acquisition phase: mice were placed in the water maze and were allowed a maximum of 60 seconds to find the submerged escape platform. Each acquisition phase day represents the mean ± SEM of three trials for each mouse in each group (n = 14 for wild type, n = 16 for mutant). (**C**) Platform reversal phase: on day 5, the position of the escape platform was moved to the opposite quadrant of the tank. Mice were placed in each of the other three quadrants and allowed a maximum of 60 seconds to find the escape platform. Results are reported as mean ± SEM.

The novel object recognition task was used to test the ability of nBmp2NLS^tm^ mice to identify familiar objects and distinguish them from novel objects^21^. In this task, mice are placed in an arena with two or three familiar objects, allowed time to explore the objects, then removed from the arena. They are subsequently placed again into the arena but with one of the now-familiar objects having been replaced by a novel object. Mice demonstrate object recognition by spending less time interacting with familiar objects and more time exploring the novel object. These tests revealed that nBmp2NLS^tm^ mutant mice spent a significantly smaller percentage of their total exploration time on the novel object (p < 0.05), suggesting decreased recognition of familiar objects (Fig. 3A).

We assessed the spatial memory capabilities of nBmp2NLS^tm^ mice compared to wild type mice using the Morris water maze^22^. In this test, mice were placed in a tank filled with opaque water containing an escape platform that was submerged approximately 1 cm below the surface. Four distinct visual cues were place on its sides to guide navigation. Mice swam three trials a day for four days, demonstrating decreased latency to the platform as they learned its location (the acquisition phase). Escape latency times improved from day 1 to day 4 and were not significantly different between wild type and mutant mice (Fig. 3B). On the fifth day, a reversal phase was performed in which the escape platform was moved to the opposite side of the tank while visual cues remained unmoved, and mice swam three trials. Mutant mice showed a trend toward slower finding of the submerged platform, but differences did not reach significance (Fig. 3C).

### Overall locomotor activity

We previously demonstrated that nBMP2 is expressed in the myonuclei of skeletal muscle and that its absence in nBmp2NLS^tm^ mice leads to slowed relaxation following stimulated twitch contraction, which may predispose mutant mice to muscle cramping during intense muscle activity^9^. Because a muscle-related reduction in overall locomotor activity could potentially skew the results of the novel object recognition task and/or the Morris water maze, we assessed overall locomotor activity in wild type and mutant mice as follows: In the Morris water maze, swimming velocity was measured during all three trials of the reversal phase. No significant differences were observed (Table 1). In the novel object recognition task, the average velocity of movement and the total distance traveled as mice moved about the arena during the acquisition phase were quantified. Again, no significant differences were detected (Table 1). Finally, the marble burying test was used to quantify digging activity^23^. No differences in digging activity were detected (Table 1). Together, these results indicate that the differences detected in the novel object recognition task cannot be attributed to differences in overall locomotor activity.

### Long-term potentiation is reduced in mature nBmp2NLS^tm^ mice

Long-term potentiation (LTP), a long-lasting enhancement in glutamatergic neurotransmission between two neurons that can also be induced by electrical stimulation *ex vivo*, is a form of synaptic plasticity thought to be the cellular equivalent of learning and memory^24^. LTP is dependent on Ca^2+^ signaling^15^,^25^,^26^. In order to measure the capacity for LTP in nBmp2NLS^tm^ mice compared to wild type mice, hippocampal brain slices were used to record LTP in CA1, the hippocampal region where nBMP2 was detected. The CA1 region is involved in spatial memory, object recognition, and other types of cognitive function^27^,^28^,^29^.

**Figure 4:**
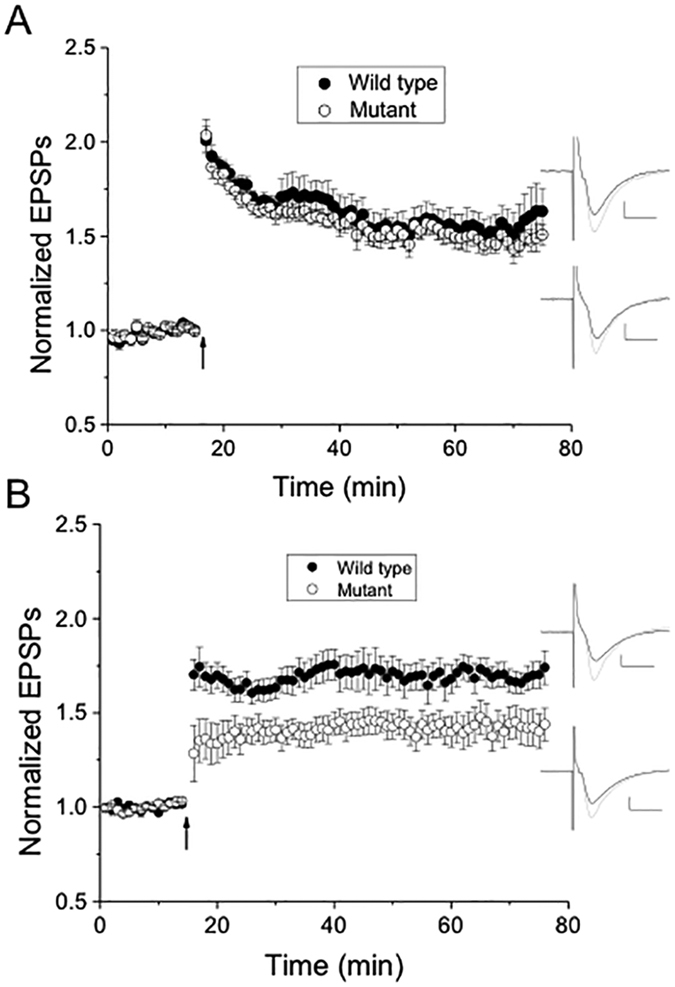
Long-term potentiation (LTP) is reduced in 3 to 4-month-old but not 1-month-old nBmp2NLS^tm^ mutant compared to wild-type mice. (**A**) In 1-month-old animals, LTP induced by theta burst (arrow) was not significantly different (p > 0.05) between nBmp2NLS^tm^ (152.1 ± 10.2%; n = 7 slices from 6 mice) and wild-type controls (158.2 ± 9.3%; n = 12 slices from 9 mice). (**B**) In 3 to 4-month-old animals, in contrast, LTP induced by theta burst (arrow) was significantly different (p < 0.05, at all 10 minute intervals post-theta burst) between nBmp2NLS^tm^ (140.1 ± 6.9%; n = 12 slices from 7 mice) and wild-type littermate control mice (172 ± 8.8%; n = 14 slices from 7 mice). Values given are for the 10-minute interval from 30–40 minutes post-conditioning. Insets in (**A**,**B**). Representative averaged excitatory postsynaptic potentials (EPSPs) that are an average of 15 individual, consecutive traces just before (black) and between 25–30 minutes after theta burst induction (gray) for both wild-type littermate and nBmp2NLS^tm^ mice. Scale bars: 10 ms, 0.25 mV.

Using 3 to 4-week-old wild type and mutant mice, we recorded field excitatory postsynaptic potentials (fEPSP) from CA1 pyramidal cells in the stratum radiatum and induced LTP with a theta-burst conditioning stimulus, which is a more natural stimulus than tetanus high frequency stimulation. Contrary to our expectation that mutant mice would have reduced LTP compared to wild type, there was no significant difference in these young mice in any of the post-conditioning 10-minute intervals (p > 0.05) (Fig. 4A).

Considering that the behavioral tests were performed on older mice, we repeated the LTP experiments in 3 to 4-month-old mice. In this population, CA1 LTP was significantly greater in wild type than in mutant mice (p < 0.05) in all of the post-conditioning 10-minute intervals (Fig. 4B). Therefore, the reduced object recognition in nBmp2NLS^tm^ mice correlates with reduced LTP, and this LTP deficit develops after the first month of life.

## Discussion

We show in this study that nBMP2 is expressed in the hippocampal CA1 pyramidal cells and that the nBmp2NLS^tm^ mouse has reduced hippocampal CA1 pyramidal cell LTP and poorer performance in the novel object recognition task, suggesting impaired object recognition memory, a function that is based in the hippocampus^17^,^19^,^30^,^31^,^32^. This data complements and expands our previous report that nBMP2 is expressed in skeletal muscle and that the nBmp2NLS^tm^ mouse shows anomalies in skeletal muscle contractions that suggest dysregulation of intracellular Ca^2+^ transport^9^. The importance of Ca^2+^ transport in hippocampal-based learning and memory formation is well documented^33^,^34^,^35^,^36^,^37^. For example, treatment of olfactory bulbectomized mice with compounds that increase the activity of Ca^2+^-responsive kinases in the hippocampus improved memory deficits and rescued impaired LTP^29^,^38^,^39^. Because an increase in postsynaptic Ca^2+^ is both necessary and sufficient to induce LTP, our finding of impaired hippocampal LTP in the nBmp2NLS^tm^ mouse again suggests that Ca^2+^ handling is disrupted in the nBmp2NLS^tm^ mouse hippocampus^15^,^40^.

The skeletal muscle and hippocampal phenotypes of the nBmp2NLS^tm^ mouse are similar to those caused by mutations in ryanodine (RyR) receptors, channel proteins that release Ca^2+^ from intracellular stores in the sarco/endoplasmic reticulum^41^,^42^. For example, ryanodine receptor 3 (RyR3) is expressed in skeletal muscle and in the hippocampal CA1 pyramidal cell layer, and RyR3 knockout mice show impairment of skeletal muscle contractions, spatial learning, and synaptic plasticity (LTP), due to the dysregulation of intracellular Ca^2+^ transport in skeletal muscle and hippocampus^43^,^44^. These phenotypic similarities reinforce the implication that nBMP2, like RyR, may play a role in intracellular Ca^2+^ transport in those cells where it is expressed.

Immunohistochemical staining revealed that nBMP2 is expressed not only in the nuclei of hippocampal CA1 pyramidal cells, but also, to a lesser extent, in the nuclei of hippocampal GABAergic interneurons that are scattered throughout the CA1 layer. The reduced LTP we observed could be caused by impaired glutamatergic signaling at synapses between CA3 Schaffer collaterals and CA1 pyramidal neurons, or alternatively it could be caused by enhanced GABAergic signaling from inhibitory neurons. Future studies are needed to settle this question. But we predict that because nBMP2 labeling was significantly less intense in GABA cells than in pyramidal cells, the effect of nBMP2 on LTP is more likely contributed by pyramidal cells.

One striking finding from this study was that the nBMP2 mutation resulted in reduced hippocampal LTP in adult but not in juvenile mice, even though immunohistochemical staining showed nBMP2 expressed in both juvenile and adult mouse hippocampus. Therefore, nBMP2 clearly plays a role in the adult hippocampus that is independent of development. The hippocampus is known to undergo significant changes during maturation from juvenile to adult, including increased expression and altered subcellular localization of plasma membrane Ca^2+^ ATPases (PMCA) and increased acetylcholine levels in the CA1 region^45^,^46^. It may be that although nBMP2 is expressed in juvenile mice, it becomes more critical for intracellular Ca^2+^ homeostasis as the hippocampus matures, accounting for the observation that nBMP2 is required for normal LTP in adult but not juvenile mice. Future studies designed to elucidate the molecular pathways linking nBMP2 with intracellular Ca^2+^ handling should shed light on this possibility.

We have demonstrated that LTP is aberrant in adult nBmp2NLS^tm^ mice. The mildly impaired object recognition memory we observed is consistent with the calcium hypothesis of brain aging, which holds that “sustained disruption of mechanisms that normally regulate intracellular Ca^2+^ signaling is pivotal for triggering adverse changes in the functioning of neurons”^47^. Sustained Ca^2+^ dysregulation in neurons has also been implicated in the cognitive decline associated with obesity, diabetes, insulin resistance/metabolic syndrome, and Alzheimer disease^48^,^49^,^50^. Additional studies are needed to fully characterize the mechanistic relationships that link nBMP2 with hippocampal LTP, but the evidence so far is consistent with our hypothesis that absence of nBMP2 from the nucleus leads to dysregulation of intracellular Ca^2+^ signaling. Once the relevant molecular pathways are fully characterized, this mouse may become a useful model for examining some aspects of the calcium hypothesis of brain aging and cognitive decline.

## Methods

### Research animals

This study was carried out in strict accordance with the recommendations in the Guide for the Care and Use of Laboratory Animals by the National Institutes of Health. The protocol was approved by the Institutional Animal Care and Use Committee (IACUC) of Brigham Young University (protocols #09-0904 and #12-0102).

Mice were housed with 5 or fewer animals per cage in a temperature-controlled (21–22 °C) room with a 12:12 hr light-dark cycle and were fed standard rodent chow and water *ad libitum*. The nBmp2NLS^tm^ mice were constructed on a Bl6/129 background, as described^9^. The homozygous wild type and mutant mice used in this study were obtained by breeding heterozygous males and females.

Mice were genotyped by PCR using the following 4 primers simultaneously: forward (GGACACCAGGTTAGTGAATCAGAACACAAG) and reverse (CTCTGACCATTATACTTCATGTGCTGGAGTTG), which bracket the site of the targeted mutation, and wtforward (CCAAACACAAACAGCGGAAGCG) and mutreverse (GGACTTGAGGGCCGCCGC), which bind to the wild type or mutant sequences respectively, at the targeted site. The forward and reverse primers produce a 909-bp product from both wild type and mutant template DNA, the forward and mutreverse primers produce a 307-bp product from mutant template DNA, and the wtforward and reverse primers produce a 634-bp product from wild type template DNA. Cycling conditions were as follows: 94 °C for 5 min; 94 °C for 20 sec, 68 °C for 30 sec, 70 °C for 40 sec, repeated 30X; 70 °C for 5 min.

### Immunohistochemistry

Immunohistochemistry was performed on brain sections from 2-month-old nBmp2NLS^tm^ and wild type mice, and on 19-day-old GAD67-GFP knock-in mice^20^. Mice were anesthetized with isoflurane and perfused transcardially with 0.9% NaCl then 4% paraformaldehyde. Brains were removed and placed in paraformaldehyde overnight, then cryoprotected in 30% sucrose before being sectioned coronally through the hippocampus at 30 μm using a cryostat. Free-floating sections were stored in 1 M PBS pH 7.4 before washing in 0.2% triton X in PBS for 30 minutes, a blocking solution of 1% bovine serum albumin and 5% normal goat serum in PBS for 2 hours, then overnight in anti-BMP2 antibody with a DyLight 650 nm fluorescent tag (Novus Biologicals, NBP1-19751C) diluted 1:100 in blocking solution. The following day the sections were washed 3 times in 1 M tris buffered saline, pH 7.4. Sections were mounted on non-frosted glass slides (Fisher Scientific), rehydrated in an ascending ethanol series, and coverslipped using DAPI Fluormount G (Southern Biotech). Images of the CA1 pyramidal cell layer were obtained using an Olympus FluoView FV1000 confocal microscope at 20x and 60x. Pictures were taken at the same laser intensity and scanning speed between groups. As a control, the antibody was preabsorbed with 10 μg/mL BMP2 peptide (Novus Biologicals, NBP1-19751PEP) for 24 hours before proceeding through the same protocol.

Quantification of immunohistochemical staining was performed using cellSens software (Olympus). To obtain grayscale intensity values for pyramidal cell nuclei, we made areas of interest of cells in the pyramidal cell layer using DAPI staining as a guide. While a few GABAergic cells may be included in this complement of cells, the vast majority are pyramidal cells. In immunohistochemistry images of GAD67-GPF mice, GABA cells were identified by green fluorescence in GFP images. In all images, areas of interest were selected that contained nuclear BMP2 without neuropil BMP2 labeling. To compare nBMP2 mean grayscale intensity between slides, background intensity levels were determined in locations where labeling was absent and used for normalization purposes. Background intensities were also subtracted to obtain the final intensity averages reported. For statistics, average nBMP2 pixel intensity from all cells of a given type in each slide were averaged in order to compare to averages for these cells in other slides using an unequal variance t test. Results are reported as the mean ± SEM.

### Novel object recognition task

The novel object recognition task^21^,^51^,^52^ was performed in a square open-field apparatus (48 cm × 48 cm × 34 cm). A total of 12 wild type and 18 mutant six-month-old male mice were used for these experiments. During the acquisition phase, mice were placed in the arena with two or three different objects and allowed to explore the objects for 10 minutes, then replaced in home cage. The arena and all objects were cleaned thoroughly to remove scent trails. After a period of 90 minutes, mice were returned to the same arena except that one of the objects had been replaced with a novel object. Mice were allowed to explore for 10 minutes. Noldus EthoVision video tracking and analysis software was used to record trials and measure the amount of time each mouse spent interacting with each object (defined as having its nose within 2 cm of the object^53^). Object recognition was calculated as time the mouse spent interacting with the novel object as a percentage of time the mouse spent interacting with all novel and familiar objects. This task was repeated seven different times on groups of mice ranging from n = 3 to n = 10, using different objects for each repeat and systematically rotating which object was used as the novel object and which was replaced with the novel object within each repeat. Results were analyzed using GraphPad Prism software to perform a paired, two-tailed t test of the seven trials. P < 0.05 was defined as statistical significance. Results were expressed as mean ± standard error of the mean (SEM).

### Morris water maze

A circular tank (114 cm diameter × 60 cm deep) was filled with water (24 °C) to a depth of ~24 cm. The water was made opaque with white non-toxic tempera paint. A circular platform (15 cm diameter × 23 cm tall) was used for the submerged escape platform. Four distinct visual cues were placed around the tank. Six-month-old male mice were used for this test, n = 14 for wild type and n = 16 for mutants. The acquisition period consisted of 3 trials per mouse per day, for 4 days. The submerged platform was placed in the northwest quadrant of the tank, and mice were released from each of the other three quadrants for their three daily trials. The order of release quadrants was rotated each day. Mice were allowed a maximum of 60 seconds to find the platform. If a mouse was unable to find the escape platform after 60 seconds, it was led to the platform and allowed to remain there for 30 seconds, and escape latency time was recorded as 60 seconds. On the 5th day, reversal phase trials were performed in which the escape platform was moved to the southeast quadrant and mice were given three trials (with release from each of the other three quadrants) to find the new position of the escape platform. Noldus Ethovision video and tracking software was used the record and analyze each trial. Statistical analyses were performed using GraphPad Prism software to perform 2-way ANOVA. For acquisition phase days, all three trials were averaged. For the reversal day, separate comparisons of genotypes within each trial were performed. Statistical significance was defined as p < 0.05. Results were expressed as mean ± SEM.

### Overall locomotor activity

Average swimming velocity was measured in the three Morris water maze reversal phase trials using Noldus Ethovision software. Average locomotion velocity and total distance traveled were measured in the acquisition phase of the novel object recognition task using Noldus Ethovison software. Digging and burying activity was measured using the marble burying test as described by Deacon using 20 marbles, with 10 and 20 minute time points^23^.

### Electrophysiological recordings and analysis

Wild type and nBmp2NLS^tm^ age-matched mice that were either 3–4 weeks old or 12–17 weeks old were anesthetized using isoflurane and decapitated. The brain was rapidly removed, and 400 μm thick coronal slices obtained using a Vibratome were stored at room temperature for at least 1 hr on a netting submerged in artificial cerebral spinal fluid (ACSF) containing 119 mM NaCl, 26 mM NaHCO_3_, 2.5 mM KCl, 1.0 mM NaH_2_PO_4_, 2.5 mM CaCl_2_, 1.3 mM MgSO_4_, and 11 mM glucose, saturated with 95% O_2_/5% CO_2_ (pH 7.4). Salts for the artificial cerebrospinal fluid were purchased from Sigma, Mallinkrodt-Baker or Fisher Scientific.

Slices were transferred to a submerged recording chamber and perfused with oxygenated ACSF (29–31 °C, pH 7.4) at a flow rate of ~2–3 ml/min for the duration of electrophysiological recordings. Average temperature for slice experiments in wild type and mutant mice were both 30 °C and not significantly different from one another. The excitatory postsynaptic potentials (EPSPs) generated at the synapse between CA3 and CA1 pyramidal cells in response to electrical stimulus were measured. Field EPSPs (fEPSPs) in CA1 were evoked at 0.1 Hz using a bipolar stainless steel electrode stimulating CA3 Schaffer collaterals, which was located 500–700 μm from a glass capillary recording electrode (~2 Mohms) filled with 1–2 M NaCl, both placed in CA1 stratum radiatum. Electrical stimulation intensity of the incoming Schaffer collaterals was adjusted to elicit a fEPSP of approximately 0.5 to 0.7 mV at the start of each experiment. Stimulus intensity ranged from between 100–200 μA for both wild type and mutant, suggesting similar AMPA receptor-mediated responses in both. Because maximal responses were approximately 1.5 to 2 mV, this means fEPSPs were adjusted to about 30–40% of maximum response. EPSPs were amplified using an Axopatch 200B (Molecular Devices), low-pass filtered at 5 kHz and sampled at 10 kHz. Signals were digitized using an Axon Digidata 1440 A (Molecular Devices) and input onto a Dell personal computer with pClamp 10.2 clampex software (Molecular Devices). LTP was elicited by a theta-burst conditioning stimulus to simulate the natural firing patterns in the brain. Two bursts were given 20 seconds apart with each burst consisting of 10 sets of 5 pulses, each pulse lasting 100 μsec and applied at 100 Hz, with 200 ms between sets. At the end of some experiments the AMPA receptor antagonist CNQX and NMDA receptor antagonist APV were applied to ensure evoked EPSPs were glutamatergic. Additional details of the electrophysiological methods have been published previously^54^.

The slope of fEPSPs was calculated using the data analysis program Clampfit 10.2 (Molecular Devices). For each individual experiment slopes from 6 stimuli per minute were averaged to create data sets in 1-minute averages. Next, all fEPSP averaged slopes for each minute were normalized to pre-conditioning fEPSP slope values (a baseline period of at least 10 min prior to electrical conditioning). Averages of fEPSP slope values in 10-minute intervals throughout post-conditioning were compared between LTP induced in wild type versus nBmp2NLS^tm^ mice using an unpaired two-tailed t-test and non-parametric Mann-Whitney test, with significance defined as p < 0.05. All combined data are expressed as the mean ± SEM.

## Additional Information

**How to cite this article**: Cordner, R. D. *et al*. The BMP2 nuclear variant, nBMP2, is expressed in mouse hippocampus and impacts memory. *Sci. Rep.* **7**, 46464; doi: 10.1038/srep46464 (2017).

**Publisher's note:** Springer Nature remains neutral with regard to jurisdictional claims in published maps and institutional affiliations.

## Figures and Tables

**Figure 1:**
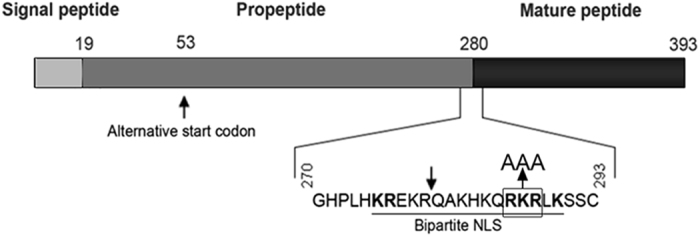
nBMP2 is synthesized by translation from a downstream alternative start codon. The BMP2 preproprotein as it is initially translated into the ER is shown schematically with the ER signal peptide (light grey), the propeptide (dark grey), and the mature peptide (black). The downward arrow indicates the site at which the BMP2 proprotein undergoes proteolytic cleavage by proprotein convertases in the Golgi to release the mature secreted peptide. Initiation at codon 53 (marked) results in cytoplasmic rather than ER translation because the ER signal peptide is absent. The protein therefore avoids proteolytic cleave and is directed to the nucleus by the bipartite nuclear localization signal (NLS) (key amino acids are in bold). The three amino acids (RKR) that were changed to alanines (AAA) to prevent nuclear localization of nBMP2 in the nBmp2NLS^tm^ mouse are shown.

**Table 1:** Mutant mice show no differences in overall locomotor activity.

	Wild type (n)	Mutant (n)	P value^e^
MWM-1^a^	12.80 (8)	12.05 (10)	0.751
MWM-2^a^	13.91 (8)	18.75 (10)	0.203
MWM-3^a^	13.89 (8)	15.22 (10)	0.614
NOR-vel^b^	4.41 (10)	4.96 (15)	0.442
NOR-dist^c^	1757.58 (10)	1898.64 (10)	0.675
MBT-10^d^	11.75 (12)	11.25 (12)	0.815
MBT-20^d^	14.58 (12)	15.33 (12)	0.696

a – average swimming velocity (cm/sec) in Morris water maze reversal phase trials 1, 2, or 3.

b – average velocity of movement (cm/sec) in acquisition phase of novel object recognition test.

c – total distance traveled (cm) in acquisition phase of novel object recognition test.

d – number of marbles buried (out of 20) in 10 or 20 minute marble burying test.

e – T-test analyses of wild type vs. mutant.
